# Does the intervention approach matter for improving 24-hour physical behaviours among overweight and obese Brazilian office workers?

**DOI:** 10.1186/s12889-025-23957-w

**Published:** 2025-08-07

**Authors:** Luiz Augusto Brusaca, Nidhi Gupta, David M. Hallman, Andreas Holtermann, Dechristian França Barbieri, Ana Beatriz Oliveira

**Affiliations:** 1 Laboratory of Clinical and Occupational Kinesiology, Department of Physical Therapy, Federal University of São Carlos, Washington Luiz Road, km 235, SP310, São Carlos, São Paulo 13565-905 Brazil; 2 National Research Centre for the Working Environment, Lersø Parkalle 105, 2100 Copenhagen, Denmark; 3 Centre for Musculoskeletal Research, Department of Occupational Health, Psychology and Sports Sciences, University of Gävle, 801 76 Gävle, Sweden; 4 Department of Industrial Engineering, Clemson University, Clemson, SC 29634 US

**Keywords:** Occupational health, Public health, Obesity, Accelerometry, 24-hour physical behaviour, Compositional data analysis

## Abstract

**Background:**

Physical behaviours over a 24-hour period are important for health. However, we do not know if interventions using a “24-hour time-use approach” are more effective in improving 24-hour time-use behaviours than the traditional “reduce sitting at work approach”. Thus, the aim of our non-randomised controlled study was to investigate this in a high-risk group of overweight and obese Brazilian office workers.

**Methods:**

Forty-five office workers were allocated to three non-randomised controlled groups; “Reduce sitting at work” (*n* = 15) receiving an intervention focused on reducing sitting time at work; the “24-hour” (*n* = 15) receiving an intervention aiming to reduce sitting at work as well as promoting behavioural changes around 24 hours (e.g., sedentary lifestyle, benefits of physical activity, and healthy sleep hygiene); or “control” (*n* = 15) without any intervention. Daily time spent in physical behaviours (sitting, standing, active, and in bed) was monitored for 7 days using a thigh-worn accelerometer at baseline, and at the 3- and 6-month follow-ups. Intervention effects were analysed using linear mixed models, adjusted for baseline values, age, and sex, with a compositional data analysis approach.

**Results:**

At baseline, the demographic characteristics and 24-hour physical behaviours of the groups were similar. No significant intervention effect was observed between the intervention groups for the overall 24-hour composition, except for time-in-bed, which decreased for Reduce sitting at work compared to 24-hour group from baseline to the 6-month follow-up (*p*-value = 0.02). Compared to the control group, both intervention approaches resulted in less time spent sitting, more time standing, and less time-in-bed from baseline to the 3-month follow-up, but these effects were not sustained at the 6-month follow-up. Notably, domain-specific (i.e., work and leisure) analysis revealed that most changes in the overall 24-hour composition occurred due to changes in behaviours during working hours.

**Conclusions:**

Among Brazilian overweight and obese office workers, the “24-hour time-use approach” may not lead to better improvements in overall 24-hour composition of physical behaviours compared to the traditional “reduce sitting at work approach”.

**Supplementary Information:**

The online version contains supplementary material available at 10.1186/s12889-025-23957-w.

## Introduction

Worldwide, obesity and extensive sedentary behaviour (e.g., sitting time) are significant public health challenges affecting individuals of all ages, ethnicities and socioeconomic groups [1–3]. Excessive body weight contributes to the development of metabolic syndrome (i.e., abdominal obesity, abnormal glycaemia, dyslipidaemia, and blood hypertension) [4]. Moreover, in combination with excessive sedentary behaviour, obese individuals have a significantly increased risk of chronic non-communicable diseases (e.g., type 2 diabetes, cardiovascular diseases, neurodegenerative diseases, and cancer) and mortality [5, 6]. This evidence highlights the potential detrimental effect of excessive weight and sedentary behaviour on health. Modifying physical behaviours, such as reducing sedentary behaviour and increasing physical activity, may contribute to weight loss (fat mass reduction) as well as mitigating health risks [7, 8]. Beyond total sedentary time, how sedentary behaviour is accumulated is important. Studies suggest that prolonged, uninterrupted bouts of sedentary behaviour are particularly harmful, whereas breaking up prolonged sedentary behaviour in shorter periods improves markers of cardiometabolic health compared to being sedentary for longer, uninterrupted periods [9, 10]. Thus, shortening the sedentary periods may, to some extent, alleviate the negative health effects of extensive prolonged sitting [7].

Among several occupational groups, office workers are well known for spending most of their work day in sedentary behaviour– i.e., sitting [11, 12]. Many also maintain their sedentariness during leisure time [11–15]. This sitting time often includes extended uninterrupted bouts of 30 minutes or more [13, 14]. Thus, several workplace interventions have been developed with the aim of reducing office workers’ sitting time, i.e., “reduce sitting at work approach”. These interventions have used strategies at different levels of workplaces to reduce sitting among the employees, such as organizational (e.g., policy changes), environmental (e.g., provision of sit-stand table), and individual (e.g., counselling) [16, 17].

Notably, most interventions were developed in high-income countries (i.e., countries belonging to the Commonwealth) [16–19], raising the question of whether they are replicable in low- and middle-income countries, such as Brazil, where workplaces often have fewer resources to support behaviour change. For instance, they do not have sit-stand tables available, and have to rely solely on health literacy and awareness (i.e., public policy) about behaviour change (e.g., reducing sitting time), which might not be enough [20]. Consequently, interventions in these contexts may need to incorporate a mix of existing workplace structures and individual strategies to encourage behaviour change. However, the effectiveness of such approaches may differ due to sociocultural factors [21]. In Brazil, for example, workplace norms, hierarchical structures, and employer-driven policies may influence workers’ ability to modify their physical behaviours, providing fewer opportunities for self-regulation compared to workplaces in high-income countries [22]. These differences highlight the need for context-specific adaptations when implementing interventions in low- and middle-income settings.

Although the “reduce sitting at work approach” has shown promising results in decreasing sitting at work [16, 18, 19], only a few studies [23–27] have targeted behaviour changes throughout the entire day, and none have focused on a 24-hour perspective, i.e., the “24-hour time-use approach”. Interventions using the entire day approach typically aimed to change physical behaviours across work and leisure domains. However, sleep is also an essential component of the day, i.e., 24-hour composition, and plays a crucial role in health and well-being. Thus, studies have shown that sleeping too much or too little may also lead to health problems, whereas a sleep duration of 7 to 8 hours per night is positively associated with health outcomes [28]. Therefore, extending interventions to change physical behaviours across the full 24-hour period aligns with the paradigm that the whole day matters, as emphasized in public health guidelines on 24-hour movement behaviour [29, 30]. Additionally, a 24-hour time-use approach may provide a more comprehensive strategy for promoting overall health and well-being [27].

To date, only a few studies using a “reduce sitting at work approach” have targeted a population of overweight and obese office workers [18, 25, 31, 32], and there is a lack of studies using the “24-hour time-use approach” in this population. Giving that this group is at an increased risk of developing metabolic syndrome and chronic non-communicable diseases [4–6], they might benefit from strategies to reduce the health risks associated with prolonged sitting, low levels of physical activity, and inappropriate sleep time. Although previous workplace interventions have focused primarily on reducing sedentary time, few have examined whether a broader, 24-hour perspective might offer additional benefits. Comparing these two approaches within the same study allows us to assess whether addressing behaviours across the full day is more effective than a workplace-specific strategy alone. Additionally, this comparison provides insight into the extent to which interventions delivered in the workplace can influence behaviours beyond working hours, an area that remains understudied.

Thus, the primary aim of this study was to evaluate the impact of a 6-month non-randomised controlled intervention on the 24-hour time-use composition of physical behaviours (i.e., sitting, standing, active, and in bed) among overweight and obese Brazilian office workers. The study compared two approaches: “Reduce sitting at work” and the “24-hour”. A control group was also included to enable investigation of the extent to which the interventions had an effect. A secondary aim was to evaluate the impact of interventions on the domain-specific (i.e., work and leisure) composition of physical behaviours.

Based on previous intervention studies on office workers [16, 19, 27], we hypothesised that both interventions would change the 24-hour time-use composition compared to controls. Specifically, we expected a reduction in sitting time and an increase in standing and active time, and no change in time-in-bed (as a proxy for sleep). However, we anticipated that the magnitude of these changes would be greater in the 24-hour group. For the domain-specific evaluation, we expected that changes in the Reduce sitting at work group would occur only during work hours, with no significant changes during leisure hours. In contrast, for the 24-hour group, we hypothesised that changes would occur both during work and leisure hours, reflecting a broader impact on daily behaviour.

## Methods

### Study design and participants

This 6-month non-randomised controlled study in real office work settings was conducted in São Carlos (São Paulo, Brazil) with a convenience sample of overweight and obese office workers employed in the administrative departments of both the Federal University of São Carlos (UFSCar) and the University Hospital of UFSCar. Data were collected between January 2021 and November 2022. The criteria for inclusion of workers in the study were: (1) being classified as overweight or obese according to body composition exam (see Measurement of anthropometric and body composition section); (2) aged 18–65 years; (3) engagement in office-based tasks (e.g., answering emails, data entry, processing documents, and browsing the internet; self-reported and confirmed during a visit by a researcher); (4) full-time work (for at least one year at the current job with plans to stay at the current job for at least 6 more months); (5) report spending most of work day sitting, i.e., > 4 hours/day; (6) no reports of chronic musculoskeletal symptoms or other chronic conditions that would preclude the use of a sit-stand table; and (7) no prior experience with sit-stand table. Given the study was conducted during the COVID-19 pandemic and the nature of the intervention, individuals working from home were not included. Pregnant workers were also not included due to the risk associated with the body composition exam.

A priori sample size calculations were performed using G*Power v.3.1.9.7 [33]. The analysis indicated that a sample size of 15 workers per group would be sufficient to detect a significant time vs. group interaction with standard probabilistic parameters (effect size f = 0.25, α = 0.05, β = 0.10). The sample size estimation was based on the statistical power for a two-tailed F-test.

Given the limited interest in participating in the study during the COVID-19 pandemic and the restricted availability of only 15 sit-stand tables equipped with reminder systems, we employed a non-randomised controlled design. This design required a structured but non-random allocation of workers into one of three groups, each comprising 15 participants. To ensure balanced group sizes and simultaneous completion of the intervention, allocation followed a rotating approach rather than being based on participant preference or ease. Furthermore, to minimize potential contamination between groups, participants who shared an office were allocated to the same group.

**Fig. 1:**
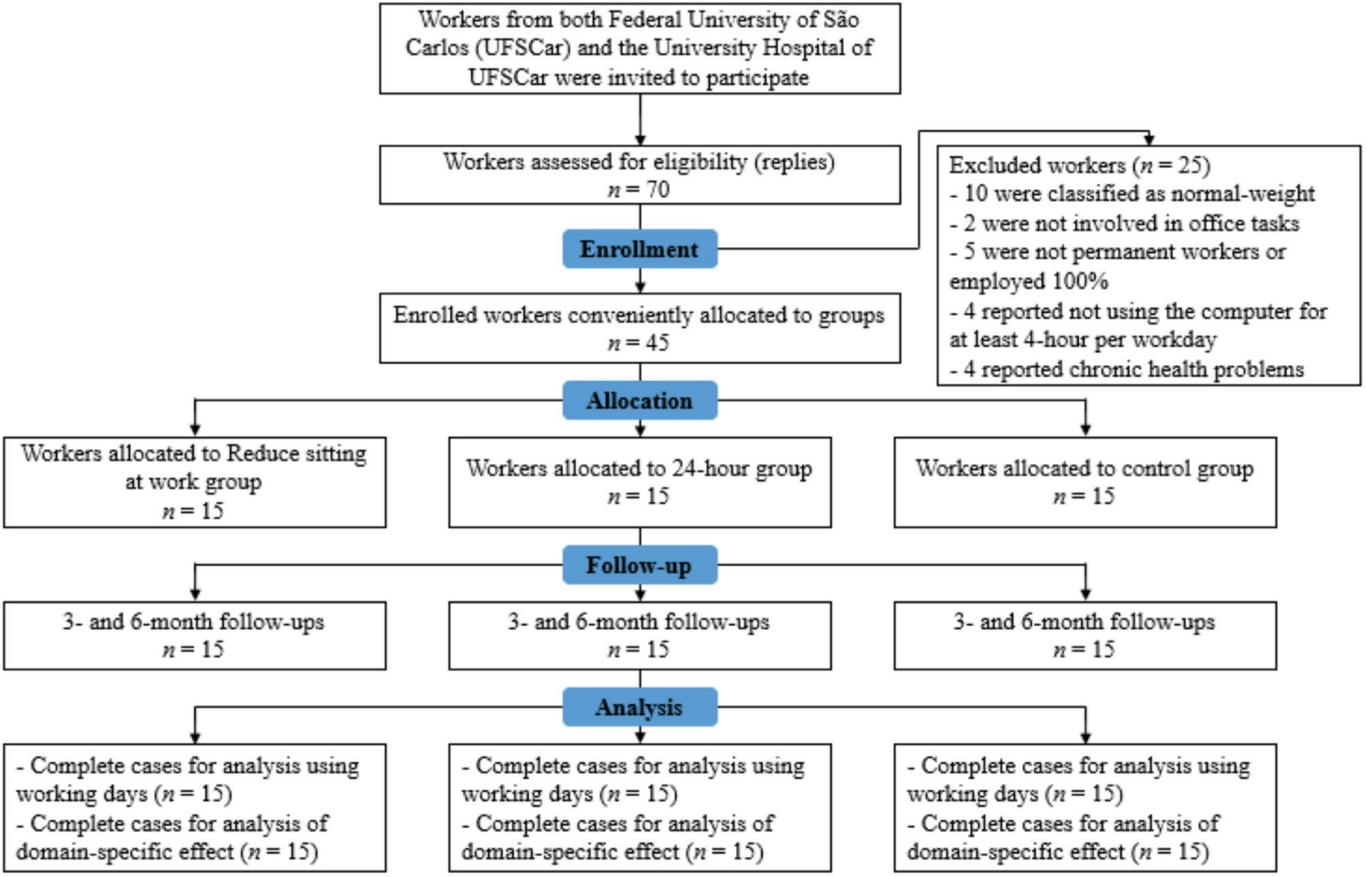
Flow chart of participant recruitment and data collection

Research staff, assessors and workers were not blinded to group allocation. All workers were invited to participate through advertisements published on the regional university’s social media. The recruitment process is illustrated in Fig. 1.

All workers provided written informed consent before joining the study. Although they received no compensation for participation, they were permitted by their employer to engage in the intervention and assessments during working hours. The study was performed in accordance with the Helsinki declaration and approved by the Human Ethics Committee of the UFSCar (São Carlos, São Paulo, Brazil; registration process #50232821.3.0000.5504). The non-randomised controlled study was also registered with the Brazilian Registry of Clinical Trials (RBR-109vwvmd). For disclosure, the registration process in the Brazilian Registry of Clinical Trials began in November 2020 but was only published in October 2022. Several factors contributed to the retrospective publication; among the most important were the priority given to the assessment of COVID-19 studies and a shortage of reviewers.

### Protocol

#### Interventions

Reduce sitting at work intervention: This intervention focused on changing the physical environment by introducing sit-stand tables equipped with a reminder system. This reminder system, a small box that emitted audio-visual alerts, was positioned on top of the worker’s table in a place that it could be seen all the time [34]. It was pre-programmed to remind the workers to change the table position after every 40 minutes of sitting (table in a low position) to 20 minutes of standing (table in a high position) [35]. The system included three buttons, allowing workers to either (i) accept, (ii) refuse, or (iii) postpone the table transition change for 2 min.

**Fig. 2:**
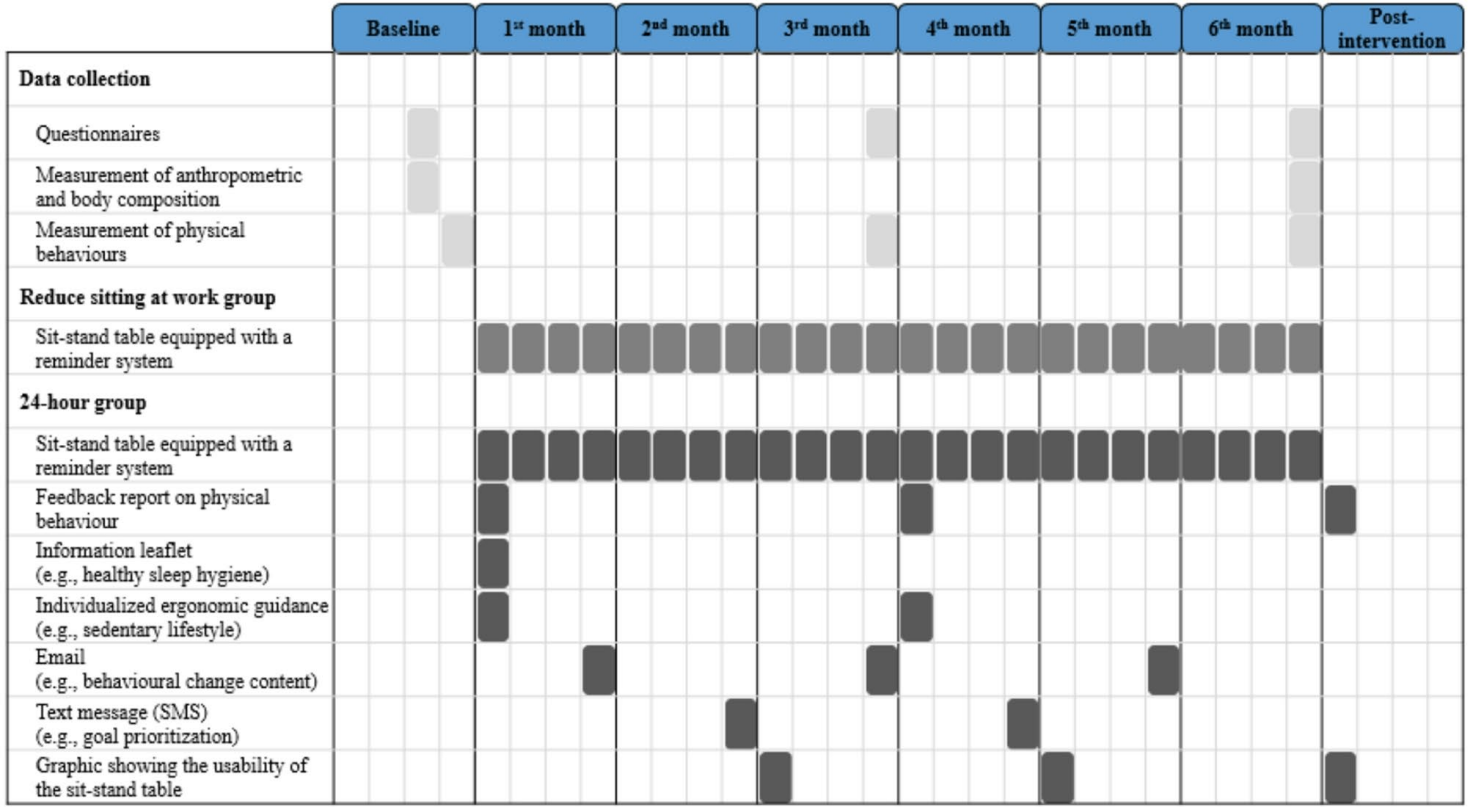
Overview of the intervention design showing environmental and individual components, as well as the chronological order in which each element was applied

24-hour intervention: This intervention used the same approach as the Reduce sitting at work intervention (i.e., change in physical environment), accompanied by an individualized behavioural change program. The individualized program was grounded in several behaviour change theories, including social cognitive theory, habit theory, self-regulation theory, and relapse prevention theory [36, 37]. It incorporated multiple Behaviour Change Techniques (BCTs), guided by the Capability, Opportunity, Motivation, and Behaviour (COM-B) model [38]. Additionally, the content of the intervention was informed by public health guidelines [29, 30] and previous evidence [27, 39–41]. The individualized program included the following components: (1) an information leaflet containing general information on sedentary lifestyle, benefits of physical activity, healthy sleep hygiene, and behavioural change [39, 40]; (2) three feedback reports on physical behaviour, provided after each assessment of physical behaviours [39, 40]; (3) two online sessions of individualized ergonomic guidance by an ergonomist, each lasting between ten to fifteen minutes [39, 40]; (4) three emails containing behavioural change content [41]; (5) two text messages with content focused on behavioural change and goal prioritization [41, 42]; and (6) three graphics illustrating the usability of the sit-stand table (e.g., the amount of time the table was in the low position) at 40, 80 and 120 days [43]. Figure 2 shows an overview of the intervention components and the chronological order in which each component was applied.

At the beginning of the intervention, workers were provided with ergonomic training from the research team on how to use the sit-stand table appropriately when in the sitting and standing positions. Workers were also asked to report the initiation of any new physical activity they undertook during the intervention period. Moreover, all workers were advised not to participate in other intervention studies or initiatives while participating in the current study.

#### Control group

Workers in the control group received no intervention and were advised to continue with their usual activities throughout the study period. After the last follow-up measurement, they were offered the opportunity to use the sit-stand table equipped with a reminder system for the same duration of the study, i.e., 6 months.

### Data collection

Data were collected at baseline and at the 3- and 6-month follow-ups (Fig. 2). Data on questionnaires, anthropometrics, body composition, and physical behaviours were obtained at all three time points, whereas anthropometric and body composition measurements were collected only at baseline and at the 6-month follow-up.

#### Demographic and personal information

The demographic and personal information questionnaire collected data on gender, age, company position (manager or employee), type of contract (permanent contract, temporary employment or fixed-term), marital status (married: yes or no), household composition (presence of children under 18 years at home: yes or no), smoking status (yes or no), and household work. Household work was assessed using a single question, “Do you perform household work?” (yes or no), and if the answer was ‘yes’, for how many minutes per day. These variables were collected to provide a more comprehensive description of the sample, which is particularly important given the non-randomised controlled design of the study.

#### Measurement of anthropometric and body composition

Height and body mass were measured on a mechanical scale (104 A; Welmy Balanças, Santa Bárbara d’Oeste, Brazil) with workers wearing light metal-free clothing (e.g., t-shirt and light trousers/skirt) and being barefoot. Height and body mass were used to calculate Body Mass Index (BMI) and to input the anthropometrics information into the Dual Energy x-ray Absorptiometry (Discovery A, Hologic, Inc., Bedford, MA, USA) device. Then, the whole-body scan of the worker was performed to obtain the Fat Mass Index (FMI; Fat Mass/Height^2^). The classification of overweight and obesity were defined according to the FMI limits of the study by Kelly et al. [44], such that an FMI > 6 kg/m^2^ classified men as overweight or obese, and an FMI > 9 kg/m^2^ classified women as such. On the day of measurements, workers were also instructed to have a light diet.

#### Measurement of physical behaviours

Physical behaviours were monitored at 20 Hz using two triaxial ActivPAL Micro 4 accelerometer (PAL technologies, Glasgow, Scotland), continuously for 24-hour a day for up to 7 days. The accelerometers were fixed with double-sided adhesive tape on the front of the worker’s right thigh, midway between the hip and the knee joint and on the upper back, at the level of the T1/T2 [45, 46]. Simultaneously, workers were asked to fill-in a diary their working hours, their time-in-bed (i.e., going to and getting out of bed to sleep), whether a day was off work, and time and reason if the accelerometer was removed. The accelerometer data were downloaded using the manufacturers’ software (PAL Software Suite Version 8) and processed using a custom-made MATLAB program, *Acti4* [47, 48], which classifies different behaviours (i.e., postures and activities) with a confirmed good validity. *Acti4* was chosen to process the data due to the possibility of combining diary information with accelerometry data and to distinguish sitting from lying using a two-sensor setup (i.e., thigh and upper back).

Using *Acti4* results, we determined three exhaustive and mutually exclusive behaviours (“compositional parts”) that completely accounted for time-use during the working day, i.e., sitting (sitting and lying), standing, and active (walking, walking stairs, running, and cycling), as done previously [49]. For the second aim of the study, which investigates domain-specific effects, these behaviours (i.e., sitting, standing, and active) were also determined for work and leisure hours separately. Time-in-bed, identified based on a diary, was added to the dataset to arrive at a full 24-hour behaviour composition. For the purpose of this study, only working days with complete 24-hour measurements and at least 4 hours of work were included for further analyses [50]. Finally, daily time spent in each behaviour was averaged over all available working days for each worker.

### Statistical analyses

Demographic and social characteristics of the workers were reported using frequencies and percentages for categorical data and means and standard deviation (SD) for continuous variables. Differences in the baseline characteristics of the workers were investigated using a one-way analysis of variance for continuous variables and a Chi-square test for categorical variables, followed by a *post hoc* multiple comparisons when significant. Continuous variables without a normal distribution were analysed using Kruskal-Wallis test. Normality was assessed using visual inspection of histograms and Q-Q plots, as well as the Shapiro-Wilk test.

Physical behaviours are inherently co-dependent and constrained within a finite 24-hour window, meaning that more time can be spent in one behaviour only at the cost of reducing time on one or more other behaviours. Because of this interdependence, traditional statistical methods are not suitable for analysing time-use data. Instead, compositional data analysis (CoDA) is recommended, as it accounts for the relative nature of the data and ensures appropriate interpretation of time-use patterns [51]. CoDA transforms the data into log-ratio coordinates, which prevent misleading statistical inferences that can arise when analysing raw time-use data. Among various log-ratio approaches, isometric log-ratio (ilr) coordinates are widely used in time-use epidemiology and allow the components of daily behaviour to be analysed within a statistically valid framework [52, 53]. For an example of CoDA implementation in occupational research, see Gupta et al. [54].

Following the CoDA approach, the daily time spent in each behaviour were expressed in terms of compositional means, in minutes (closed to a total duration of 1440 min, i.e., 24-hour), as well as percentages (closed to 100%). Then, the dispersion between the behaviours was calculated using the variation matrix, which measures the variance of the logarithms of all possible pair-wise ratios (e.g., variance of ln(Sit/Stand)). Furthermore, the 24-hour behaviour compositions of working days at each measurement point (i.e., baseline, 3- and 6-month follow-ups) were transformed into sets of three orthogonal ilr-coordinates [55], as exemplified below for sitting:

$$ \begin{gathered} \:{\text{ilr}}_{1} = \sqrt {\frac{3}{4}} \hfill \,ln\left( {\frac{{Sit}}{{\sqrt[3]{{Stand\:*\:Active\:*\:Time - in - bed}}}}} \right) \hfill \\ \end{gathered} $$

$$\:{\text{i}\text{l}\text{r}}_{2}=\sqrt{\frac{2}{3}}{ln}\left(\frac{Stand}{\sqrt[2]{Active\:*\:Time-in-bed}}\right)$$

$$\:{\text{i}\text{l}\text{r}}_{3}=\sqrt{\frac{1}{2}}{ln}\left(\frac{Active}{Time-in-bed}\right)$$

In this case, ilr_1_ expresses the ratio of time spent sitting to time spent in all other behaviours. For each of the four behaviours, a separate set of three ilr-coordinates was created by rotating the position of behaviours in the equation. This allowed each behaviour to be expressed in relation to the remaining behaviours [55]. To address the second aim concerning domain-specific effects, the 24-hour behaviour compositions for work and leisure hours were transformed into sets of six ilr-coordinates. Adopting the previously described rotating principle, a unique set of six ilr-coordinates was created for each of the seven behaviours, thus expressing each behaviour in relation to all others. The equations pertaining to the second aim are noted in the Additional file 1. This ilr transformation allows data to be analysed further using standard statistical methods [53, 54].

After applying the ilr transformation to the time-use data, changes from baseline to the 3- and 6-month follow-up were calculated. Separate linear mixed models were fitted with the change in each of the first ilr of each set of ilr-coordinates to examine the extent to which the 24-hour time-use composition differed between the intervention groups and the control group at the 3- and 6-month follow-ups. The models included random intercepts to account for the within-participant clustering of repeated measures and estimated fixed effects for time (2 levels), group (3 levels), and their interaction (2 vs. 3 levels). The models were adjusted for baseline values of the outcome, age and sex. An unstructured within-participant covariance was used in the models to deal with the repeated measures (3- and 6-month follow-up). The statistical model used for analysing the change in the first ilr-coordinate was defined as follows:

$$ \begin{gathered} \Delta {\text{ilr}}_{{1,ij}} = ~\beta _{0} + ~\beta _{1} Time_{{ij}} + ~\beta _{2} Group_{{ij}} \hfill \\ + ~\beta _{3} \left( {Time~x~Group} \right)_{{ij}} + ~\beta _{4} Baseline~{\text{ilr}}_{{1,i}} \hfill \\ + ~\beta _{5} Age_{i} + ~\beta _{6} Sex_{i} + ~u_{i} +{\epsilon}_{{ij}} \hfill \\ \end{gathered} $$

where $\:{\varDelta\:ilr}_{1,ij}$ represents the change in the first ilr-coordinate for participant *i* at time point *j*.

Estimates of within-group changes and between-group differences were obtained using marginal means and pairwise comparisons of marginal means. The normal distribution of residuals was visually inspected *post hoc* and confirmed that the normality assumptions for linear regression were fulfilled.

All statistical analyses were carried out using the software R v4.3.3 [56], CoDA data processing were performed using the package ‘compositions’ v2.0-2 [57] and linear mixed models were performed using the ‘nlme’ v3.1-166 package [58].

## Results

### Flow of participants

The flow of participants is illustrated in Fig. 1. Out of a total of 70 office workers who expressed interest in participating, 45 were enrolled in the study. Throughout the follow-up measurements, no participants withdrew, and all agreed to complete all measurements.

### Baseline characteristics

Baseline demographic and social characteristics for all workers and for each intervention group are shown in Table 1. The sample consisted of slightly more men than women, with a mean age of 39.0 (SD 8.3) years. Most workers had an employee position at the company, were married, had children under 18 years old living at home, were non-smokers, and engaged in household work for an average of 72.1 (SD 36.3) min per day. The average BMI was 28.8 (SD 4.4) kg/m^2^ and the average FMI for women was 12.3 (SD 3.8) kg/m^2^ and 9.1 (SD 2.4) kg/m^2^ for men. Based on FMI thresholds, most workers (68.2%) were classified as overweight. There were no marked differences in any of the demographic characteristics between the groups (Table 1).

All workers had valid baseline accelerometer data (i.e., including only days with complete 24-hour measurements and at least 4 hours of work), as well as at the 3- and 6-month follow-ups. The description of the accelerometer data is presented in the Additional file 2, Table S1.

**Table 1:** Baseline demographic and social characteristics of participants conveniently allocated to the Reduce sitting at work group (intervention with physical environment component), 24-hour group (intervention with physical environment and individual component), and control group

	All workers	Reduce sitting at work	24-hour	Control	*p*-value
Sample, n	45	15	15	15	
Gender, n (%)^a^					0.45
Women	21 (46.7)	8 (53.3)	5 (33.3)	8 (53.3)	
Men	24 (53.3)	7 (46.7)	10 (66.7)	7 (46.7)	
Age (years), mean (SD)^a^	39.0 (8.3)	37.4 (10.0)	40.0 (7.7)	39.7 (7.2)	0.65
Company position, n (%)^a^					0.53
Manager	19 (42.2)	8 (53.3)	6 (40.0)	5 (33.3)	
Employee	26 (57.8)	7 (46.7)	9 (60.0)	10 (66.7)	
Married (yes), n (%)^a^	33 (73.3)	12 (80.0)	12 (80.0)	9 (60.0)	0.36
Children at home (yes), n (%)^a^	24 (53.3)	9 (60.0)	8 (53.3)	7 (46.7)	0.77
Smokers (yes), n (%)^a^	4 (8.9)	3 (20.0)	0 (0.0)	1 (6.7)	0.15
Household work^a^					
Perform (yes), n (%)	38 (84.4)	14 (93.3)	11 (73.3)	13 (86.7)	0.31
Minutes per day, mean (SD)	72.1 (36.3)	68.6 (29.8)	76.4 (38.8)	72.3 (42.7)	0.86^c^
Height (m), mean (SD)^b^	1.70 (0.07)	1.69 (0.09)	1.68 (0.06)	1.72 (0.06)	0.27^c^
Weight (kg), mean (SD)^b^	83.1 (15.4)	85.7 (18.4)	80.0 (13.2)	83.7 (14.7)	0.60^c^
BMI (kg/m^2^), mean (SD)^b^	28.8 (4.4)	29.8 (4.2)	28.4 (4.5)	28.3 (4.5)	0.60^c^
FMI (kg/m^2^), mean (SD)^b^					
Women	12.3 (2.8)	12.2 (1.7)	12.8 (3.6)	12.0 (3.5)	0.91^c^
Men	9.1 (2.4)	9.7 (3.0)	9.2 (2.3)	8.4 (2.2)	0.63^c^
All workers	10.6 (3.0)	11.0 (2.6)	10.4 (3.2)	10.2 (3.4)	0.76^c^
FMI classification, n (%)^b^					0.34
Overweight	30 (66.7)	9 (60.0)	10 (66.7)	11 (73.3)	
Obese class 1	10 (22.2)	5 (33.3)	3 (20.0)	2 (13.3)	
Obese class 2	4 (8.9)	0 (0.0)	2 (13.3)	2 (13.3)	
Obese class 3	1 (2.2)	1 (6.7)	0 (0.0)	0 (0.0)	

*n* number of workers, *SD* standard deviation, *BMI* Body Mass Index, *FMI* Fat Mass Index

*p*-values refer to tests of the differences between groups

^a^Self-reported information from the questionnaire

^b^Directly measured

^c^Non-normally distributed variable. Kruskal-Wallis test was used for between-group comparison. Normality was visually evaluated using histograms and Q–Q plots and tested using the Shapiro-Wilk test (both results not shown)

### Physical behaviour composition

Table 2 shows the compositional mean values of time spent sitting, standing, active and in bed at each measurement point (i.e., baseline, 3- and 6-month follow-ups) for the intervention groups and the control group, for the overall 24-hour composition and specifically for the 24-hour domain-specific (i.e., work and leisure) composition. The overall 24-hour composition of the intervention groups changed throughout the intervention period. Specifically, the Reduce sitting at work group and the 24-hour group spent less time sitting and more time standing (Table 2). The 24-hour domain-specific composition showed that most changes in sitting and standing occurred during work hours rather than during leisure hours. The variation matrix, which indicates the dispersion of each behaviour relative to other behaviours, is presented in the Additional file 3, Tables S1 and S2. In general, sitting and time-in-bed were the most proportional behaviours, i.e., values of variation matrix close to zero. The total variance of the behaviours (i.e., the total relative variability of the time-use composition) increased from baseline to the 3- and 6-month follow-ups; standing contributed most to this change in total variance.

**Table 2:** Baseline, 3- and 6-month follow-up values for minutes spent sitting, standing, active, and in bed, representing the overall 24-hour composition, as well as 24-hour domain-specific (i.e., work and leisure) composition

Measurement	Behaviours	Reduce sitting at work	24-hour	Control
Overall 24-hour composition				
Baseline	Sitting	726 (50.4)	745 (51.7)	709 (49.2)
	Standing	156 (10.8)	141 (9.8)	150 (10.4)
	Active	125 (8.7)	113 (7.9)	133 (9.3)
	Time-in-bed	434 (30.1)	441 (30.6)	448 (31.1)
3-month	Sitting	699 (48.5)	682 (47.4)	727 (50.5)
	Standing	190 (13.2)	199 (13.8)	139 (9.6)
	Active	131 (9.1)	119 (8.3)	121 (8.4)
	Time-in-bed	421 (29.2)	440 (30.5)	452 (31.4)
6-month	Sitting	736 (51.1)	712 (49.4)	714 (49.6)
	Standing	169 (11.7)	155 (10.8)	152 (10.6)
	Active	120 (8.3)	104 (7.3)	127 (8.8)
	Time-in-bed	415 (28.8)	469 (32.5)	446 (31.0)
24-hour domain-specific (i.e., work and leisure) composition				
Baseline	*Work*			
	Sitting	429 (29.8)	469 (32.6)	439 (30.5)
	Standing	57 (4.0)	54 (3.8)	58 (4.0)
	Active	50 (3.5)	45 (3.1)	53 (3.7)
	*Leisure*			
	Sitting	298 (20.7)	280 (19.5)	267 (18.6)
	Standing	96 (6.7)	79 (5.5)	90 (6.3)
	Active	74 (5.1)	65 (4.5)	80 (5.6)
	Time-in-bed	436 (30.3)	446 (31.0)	452 (31.4)
3-month	*Work*			
	Sitting	392 (27.2)	388 (26.9)	441 (30.6)
	Standing	93 (6.5)	115 (8.0)	55 (3.8)
	Active	53 (3.7)	54 (3.7)	50 (3.5)
	*Leisure*			
	Sitting	310 (21.5)	296 (20.6)	283 (19.7)
	Standing	91 (6.4)	81 (5.6)	83 (5.8)
	Active	76 (5.3)	63 (4.4)	71 (4.9)
	Time-in-bed	424 (29.5)	443 (30.8)	457 (31.7)
6-month	*Work*			
	Sitting	413 (28.7)	420 (29.2)	448 (31.1)
	Standing	85 (5.9)	85 (5.9)	61 (4.2)
	Active	55 (3.8)	44 (3.0)	54 (3.8)
	*Leisure*			
	Sitting	324 (22.5)	293 (20.3)	265 (18.4)
	Standing	79 (5.5)	67 (4.7)	90 (6.2)
	Active	63 (4.4)	58 (4.1)	72 (5.0)
	Time-in-bed	421 (29.2)	472 (32.8)	450 (31.2)

Values are presented as compositional mean in minutes scaled, to a total duration of 1440 min (i.e., 24-hour), with percentage of time in parentheses

Reduce sitting at work group: intervention with physical environment component; 24-hour group: intervention with physical environment and individual component; control group: no intervention provided

### Intervention effects on overall and domain-specific 24-hour time-use composition

Tables 3 and 4 show the mean ilr-coordinates of each behaviour at baseline by group, the adjusted mean change from baseline to the 3- and 6-month follow-ups, and the adjusted mean difference between groups at each follow-up, as obtained from the linear mixed models. Table 3 provides these values for the overall 24-hour composition, while Table 4 focuses on the 24-hour domain-specific (i.e., work and leisure) composition.

#### Overall 24-hour composition

Reduce sitting at work group vs. the 24-hour group: No significant intervention effects (i.e., change from baseline) were observed on sitting, standing, and active behaviour between the Reduce sitting at work group and the 24-hour group at the 3- or 6-month follow-ups (Table 3). For time-in-bed, no intervention effect were observed at 3-month follow-up, but at the 6-month follow-up, the Reduce sitting at work group spent significantly less time-in-bed relative to all other behaviours compared to the 24-hour group (adjusted mean difference in logarithmic units: − 0.16 [95% confidence interval (CI) − 0.30; − 0.02], *p*-value = 0.02).

Reduce sitting at work group vs. control group: No significant intervention effects were observed on sitting between the Reduce sitting at work group and the control group at the 3- or 6-month follow-ups (Table 3). For standing time, the intervention effects at the 3-month follow-up showed that the Reduce sitting at work group spent more time standing relative to all other behaviour compared to the control group (0.25 [95%CI 0.03; 0.46], *p*-value = 0.02). No significant intervention effects were observed for active behaviour in the Reduce sitting at work group compared to the control group at the 3- or 6-month follow-ups (Table 3). Time-in-bed was reduced at the 3-month follow-up in the Reduce sitting at work group compared to the control group (– 0.16 [95%CI − 0.29; − 0.02], *p*-value = 0.02). By the 6-month follow-up, this effect was no longer observed (Table 3).

24-hour group vs. control group: At the 3-month follow-up, the 24-hour group spent less time sitting relative to all other behaviour compared to the control group (– 0.23 [95%CI − 0.41; − 0.04], *p*-value = 0.01). At the 6-month follow-up, the intervention effects on sitting time weakened and were no longer significant (Table 3). For standing time, the intervention effects at the 3-month follow-up showed that the 24-hour group spent more time standing relative to all other behaviour compared to the control group (0.35 [95%CI 0.13; 0.56], *p*-value < 0.01). No significant intervention effects were observed for active behaviour and time-in-bed in the 24-hour group compared to the control group at the 3- or 6-month follow-ups (Table 3).

#### Domain-specific composition

Reduce sitting at work group vs. the 24-hour group: No significant intervention effects were observed during work and leisure hours between the Reduce sitting at work group and the 24-hour group at the 3- or 6-month follow-ups (Table 4), except for time-in-bed. At the 6-month follow-up, the Reduce sitting at work group spent significantly less time-in-bed relative to all other behaviours compared to the 24-hour group (– 0.16 [95%CI − 0.31; − 0.01], *p*-value = 0.04).

Reduce sitting at work group vs. control group: During work hours at the 3-month follow-up, the Reduce sitting at work group spent less time sitting relative to all other behaviours compared to the control group (– 0.22 [95%CI − 0.42; − 0.02], *p*-value = 0.03). This effect became weaker and non-significant at the 6-month follow-up. For standing time during work hours, the intervention effects at the 3- and 6-month follow-ups showed that the Reduce sitting at work group spent more time standing relative to all other behaviour compared to the control group (Table 4). No significant intervention effects were observed for active behaviour during work hours or for sitting, standing and active behaviour during leisure hours in the Reduce sitting at work group compared to the control group (Table 4). For time-in-bed, reductions at the 3-month follow-up were observed in the Reduce sitting at work group compared to the control group (– 0.17 [95%CI − 0.32; − 0.03], *p*-value = 0.02).

24-hour group vs. control group: During work hours at the 3-month follow-up, the 24-hour group spent less time sitting relative to all other behaviour compared to the control group (– 0.32 [95%CI − 0.53; − 0.12], *p*-value < 0.01). By the 6-month follow-up, this effect was no longer observed (Table 4). For standing time during work hours, the 24-hour group spent more time standing relative to all other behaviour compared to the control group at both the 3- and 6-month follow-ups (Table 4). No significant intervention effects were observed for active behaviour during work hours, for sitting, standing and active behaviour during leisure hours, and for time-in-bed in the 24-hour group compared to the control group (Table 4).

## Discussion

The aim of this study was to investigate whether an intervention using a “24-hour time-use approach” was more effective in improving the overall 24-hour composition of physical behaviours than the traditional “reduce sitting at work approach” in a sample of overweight and obese Brazilian office workers, as well as to assess the interventions effect compared to a control group. Additionally, we examined the effects of the interventions on the domain-specific (i.e., work and leisure) composition of physical behaviours. In line with our hypothesis, both intervention groups demonstrated changes in their overall 24-hour behaviours compared to the control group, with reductions in sitting time and increases in standing time. However, contrary to our expectations, the magnitude of these changes was not greater in the 24-hour group, and no significant improvements were observed in active time or time-in-bed. Furthermore, as hypothesised, changes in the Reduce sitting at work group were largely restricted to work hours, while the 24-hour group did not show significant changes during leisure hours.

**Table 3:** Intervention effects on changes from baseline to the 3- and 6-month follow-ups in the overall 24-hour composition of physical behaviours, adjusted for baseline values of the outcome, age and sex

Behaviours	Time	Mean (SD) isometric log-ratio at baseline^a^			Adjusted mean [95% CI] change from baseline to follow-up^b^			Adjusted mean difference at follow-up [95% CI]^c^		
		Reduce sitting at work	24-hour	Control	Reduce sitting at work	24-hour	Control	Reduce sitting at work (ref) vs. 24-hour	Reduce sitting at work (ref) vs. Control	24-hour (ref) vs. Control
Sitting	3-month	1.10 (0.25)	1.18 (0.22)	1.06 (0.16)	–0.10 [–0.21; 0.00]	–0.17 [–0.28; −0.06]	0.06 [–0.04; 0.17]	0.07 [–0.12; 0.25]	–0.16 [–0.34; 0.02]	**–0.23** **[–0.41; −0.04]**
	6-month				0.00 [–0.10; 0.11]	–0.04 [–0.15; 0.07]	0.01 [–0.10; 0.12]	0.04 [–0.14; 0.23]	–0.01 [–0.19; 0.17]	–0.05 [–0.23; 0.13]
Standing	3-month	–0.68 (0.24)	–0.75 (0.24)	–0.73 (0.18)	0.20 [0.07; 0.32]	0.30 [0.17; 0.42]	–0.05 [–0.18; 0.07]	–0.10 [–0.32; 0.12]	**0.25** **[0.03; 0.46]**	**0.35** **[0.13; 0.56]**
	6-month				0.11 [–0.02; 0.24]	0.09 [–0.04; 0.22]	0.02 [–0.10; 0.15]	0.02 [–0.20; 0.24]	0.09 [–0.13; 0.30]	0.06 [–0.15; 0.28]
Active	3-month	–0.93 (0.26)	–1.00 (0.17)	–0.87 (0.23)	0.01 [–0.08; 0.10]	–0.06 [–0.15; 0.03]	–0.05 [–0.14; 0.04]	0.07 [–0.08; 0.22]	0.06 [–0.09; 0.21]	–0.01 [–0.16; 0.14]
	6-month				–0.04 [–0.13; 0.05]	–0.13 [–0.22; −0.04]	–0.03 [–0.11; 0.06]	0.09 [–0.06; 0.24]	–0.01 [–0.16; 0.13]	–0.11 [–0.26; 0.05]
Time-in-bed	3-month	0.51 (0.24)	0.57 (0.17)	0.54 (0.18)	–0.11 [–0.19; −0.03]	–0.07 [–0.15; 0.01]	0.05 [–0.03; 0.13]	–0.04 [–0.18; 0.10]	**–0.16** **[–0.29; −0.02]**	–0.12 [–0.26; 0.02]
	6-month				–0.08 [–0.16; 0.00]	0.08 [0.00; 0.16]	0.00 [–0.07; 0.08]	**–0.16** **[–0.30; −0.02]**	–0.08 [–0.22; 0.06]	0.08 [–0.06; 0.21]

^a^ Behaviour expressed in terms of the first isometric log-ratio coordinate (ilr_1_), i.e., representing the time spent in that behaviour relative to time spent in all other behaviours. A positive ilr value indicates that time spent in the numerator behaviour (e.g., sitting) was larger than time spent in the denominator behaviour (e.g., all other behaviours), while a negative ilr value indicates the opposite

^b^ Adjusted means are estimated from marginal means at the 3- and 6-month follow-ups, with baseline values of the outcome, age and sex set to their overall mean. A positive value indicates an increase in time spent in a given behaviour relative to baseline values, while negative values indicate a decrease

^c^ Adjusted mean difference between groups are estimated from pairwise comparisons and contrasts of marginal means at the 3- and 6-month follow-ups, with baseline values of the outcome, age and sex set to their overall mean. A positive value indicates that the reference group (ref) increased the time spent on a given behaviour compared to the other group, while negative values indicate a decrease

Bolded text indicates a statistically significant intervention effect (i.e., *p*-value < 0.05)

**Table 4:** Intervention effects on changes from baseline to 3- and 6-month follow-ups in the 24-hour domain-specific (i.e., work and leisure) composition of physical behaviours, adjusted for baseline values of the outcome, age and sex

Behaviours		Time	Mean (SD) isometric log-ratio at baseline^a^			Adjusted mean [95% CI] change from baseline to follow-up^b^			Adjusted mean difference at follow-up [95% CI]^c^		
			Reduce sitting at work	24-hour	Control	Reduce sitting at work	24-hour	Control	Reduce sitting at work vs. 24-hour	Reduce sitting at work vs. Control	24-hour vs. Control
Work	sitting	3-month	1.20 (0.28)	1.36 (0.28)	1.22 (0.24)	–0.19 [–0.30; −0.07]	–0.29 [–0.41; −0.17]	0.03 [–0.08; 0.15]	0.10 [–0.11; 0.31]	**–0.22** **[–0.42; − 0.02]**	**–0.32** **[–0.53; 0.12]**
		6-month				–0.09 [–0.20; 0.03]	–0.10 [–0.22; 0.02]	0.02 [–0.10; 0.13]	0.02 [–0.19; 0.23]	–0.10 [–0.30; 0.10]	–0.12 [–0.32; 0.09]
	standing	3-month	–0.98 (0.34)	–0.97 (0.56)	–0.98 (0.36)	0.48 [0.28; 0.68]	0.67 [0.47; 0.87]	–0.02 [–0.22; 0.17]	–0.19 [–0.53; 0.15]	**0.50** **[0.16; 0.84]**	**0.69** **[0.35; 1.03]**
		6-month				0.43 [0.23; 0.63]	0.44 [0.24; 0.64]	0.06 [–0.13; 0.26]	-0.01 [-0.35; 0.33]	**0.37** **[0.03; 0.70]**	**0.38** **[0.04; 0.72]**
	active	3-month	–1.12 (0.25)	–1.17 (0.42)	–1.06 (0.28)	–0.02 [–0.16; 0.12]	0.03 [–0.11; 0.17]	–0.01 [–0.15; 0.13]	-0.05 [-0.29; 0.19]	–0.02 [–0.25; 0.22]	0.04 [–0.20; 0.27]
		6-month				0.08 [–0.06; 0.22]	–0.09 [–0.23; 0.05]	0.05 [–0.09; 0.18]	0.17 [–0.07; 0.41]	0.03 [–0.20; 0.27]	–0.14 [–0.37; 0.10]
Leisure	sitting	3-month	0.80 (0.25)	0.80 (0.26)	0.68 (0.25)	–0.02 [–0.16; 0.11]	–0.03 [–0.17; 0.11]	0.07 [–0.06; 0.21]	0.01 [–0.22; 0.24]	–0.10 [–0.33; 0.13]	–0.10 [–0.34; 0.13]
		6-month				0.07 [–0.06; 0.21]	0.05 [–0.08; 0.19]	–0.03 [–0.17; 0.10]	0.02 [–0.21; 0.25]	0.10 [–0.13; 0.33]	0.09 [–0.14; 0.32]
	standing	3-month	–0.42 (0.28)	–0.56 (0.41)	–0.49 (0.20)	–0.09 [–0.24; 0.06]	–0.12 [–0.27; 0.03]	–0.05 [–0.20; 0.10]	0.04 [–0.23; 0.30]	–0.03 [–0.29; 0.22]	–0.07 [–0.33; 0.19]
		6-month				–0.20 [–0.36; −0.05]	–0.23 [–0.38; −0.07]	–0.01 [–0.16; 0.14]	0.02 [–0.24; 0.28]	–0.20 [–0.45; 0.06]	–0.22 [–0.48; 0.04]
	active	3-month	–0.70 (0.29)	–0.78 (0.31)	–0.62 (0.24)	–0.02 [–0.16; 0.13]	–0.20 [–0.35; −0.05]	–0.05 [–0.20; 0.09]	0.18 [–0.07; 0.43]	0.04 [–0.21; 0.28]	–0.14 [–0.40; 0.11]
		6-month				–0.18 [–0.33; −0.04]	–0.18 [–0.33; −0.03]	–0.07 [–0.21; 0.08]	0.00 [–0.25; 0.25]	–0.12 [–0.36; 0.13]	–0.12 [–0.37; 0.14]
time-in-bed		3-month	1.22 (0.25)	1.31 (0.20)	1.25 (0.20)	–0.12 [–0.21;–0.04]	–0.10 [–0.18; −0.01]	0.05 [–0.04; 0.13]	–0.03 [–0.17; 0.12]	**–0.17** **[–0.32; −0.03]**	–0.14 [–0.29; 0.00]
		6-month				–0.09 [–0.17; 0.00]	0.07 [–0.02; 0.16]	0.00 [–0.09; 0.08]	**–0.16** **[–0.31; −0.01]**	–0.09 [–0.23; 0.06]	0.07 [–0.07; 0.22]

^a^ Behaviour expressed in terms of the first isometric log-ratio coordinate (ilr_1_), i.e., representing the time spent in that behaviour relative to time spent in all other behaviours. A positive ilr value indicates that time spent in the numerator behaviour (e.g., sitting) was larger than time spent in the denominator behaviour (e.g., all other behaviours), while a negative ilr value indicates the opposite

^b^ Adjusted means are estimated from marginal means at the 3- and 6-month follow-ups, with baseline values of the outcome, age and sex set to their overall mean. A positive value indicates an increase in time spent in a given behaviour relative to baseline values, while negative values indicate a decrease

^c^ Adjusted mean difference between groups are estimated from pairwise comparisons and contrasts of marginal means at the 3- and 6-month follow-ups, with baseline values of the outcome, age and sex set to their overall mean. A positive value indicates that the reference group (ref) increased the time spent on a given behaviour compared to the other group, while negative values indicate a decrease

Bolded text indicates a statistically significant intervention effect (i.e., *p*-value < 0.05)

No intervention effect was observed between the Reduce sitting at work group vs. the 24-hour group in their overall 24-hour composition or on the domain-specific (i.e., work and leisure) composition of physical behaviours at the 3- or 6-month follow-ups, except for time-in-bed, which decreased in the Reduce sitting at work group compared to the 24-hour group from baseline to the 6-month follow-up. Compared to the control group, both intervention approaches were shown to be effective in changing the overall 24-hour behaviours. Specifically, there was a reduction in sitting time and an increase in standing time. These findings partially align with our hypothesis, as changes were observed primarily during work hours, reinforcing the idea that workplace-based interventions have limited influence on leisure-time behaviours.

Our results align with previous workplace studies using similar interventions with sit-stand table, where sitting was primarily replaced with standing, with small but possibly systematic increases in active behaviour among overweight and obese office workers [25, 31, 32], as well as in the general population [16, 18, 19, 27, 59]. Although our results are consistent with the literature, our approach to processing the physical behaviour data using the CoDA method makes comparison with previous studies somewhat challenging, as these studies did not account for the full 24-hour composition (e.g., sitting, standing, active, and sleep) and did not address the compositional nature of physical behaviours [53, 54].

To our knowledge, only two studies [32, 60] have evaluated the effects of a workplace intervention targeting physical behaviour with data processed using the CoDA approach. For example, the study by Larisch et al. [60] evaluated the effect of two multi-component interventions using a combination of cognitive behavioural therapy and motivational interviewing in office workers who already had access to sit-stand tables. They found no significant intervention effect (i.e., interaction between time and group) on the overall 24-hour compositions or on the domain-specific compositions. Similarly, the study by Barbieri et al. [32] aimed to compare whether the use of sit-stand table over 6-month similarly affected the physical behaviours of normal-weight and overweight office workers. They found that, during working hours, both groups decrease sitting time in favour of more time standing, with normal-weight workers exhibiting a greater extent of behaviour change. During leisure hours, no effect of the intervention was observed, confirming that there are no significant compensatory effects on physical behaviours due to the implementation of the intervention at work, which corroborates with previous studies [27, 31, 60].

Our results for the domain-specific compositions of the Reduce sitting at work group corroborate with these findings, as no intervention effect was observed for leisure behaviours (cf. Table 4). This supports our hypothesis that behaviour change in this group would be limited to work hours. Although no compensatory effect was observed for the 24-hour group, there was also no improvement in leisure behaviours, suggesting that interventions delivered at the workplace may be insufficient to drive meaningful changes during leisure hours, even when focused on promoting behaviour change across the entire day [23, 24, 27]. More research is needed to understand how to effectively support behaviour change during leisure hours.

### The lack of effect between the intervention approaches

Interestingly, our study observed no significant advantage for the “24-hour time-use approach” over the “reduce sitting at work approach” in improving overall 24-hour composition of physical behaviours. This lack of difference may suggest that, despite the additional components aimed at promoting behavioural change (cf. Figure 2), the 24-hour approach did not lead to substantial changes during leisure time, where participants have more autonomy over their behaviours and environmental constraints differ from the workplace. One possible explanation is that leisure time behaviours are less structured and more influenced by personal preferences, social norms, and home environments, making them less responsive to workplace-based interventions [26, 27, 61]. Even though participants in the 24-hour group were provided with materials, feedback, and individualized ergonomic guidance on broader health and lifestyle behaviours, these strategies may not have been sufficient to overcome the personal and environmental barriers that shape leisure time behaviours. Additionally, the strategies used in this intervention may not have been intensive, frequent, or interactive enough to sustain long-term changes. Future interventions could benefit from incorporating more real-time engagement strategies, such as mobile app reminders or activity tracking feedback, to better support sustained behaviour change.

Participants were selected by convenience using a non-randomised controlled design, which introduces the potential for sampling and self-selection bias [62] and may have influenced results based on each participant’s motivation and willingness to engage with behaviour change strategies [63]. For instance, some participants in the 24-hour group may have joined the study primarily to use the sit-stand table to modify work hour behaviours, even though they also received guidance to encourage behaviour change during leisure hours. Additionally, competing demands, personal habits, and family responsibilities could have further limited behaviour change outside of working hours. Another contributing factor may be the small sample size, which limits the ability to detect smaller effect sizes and may have reduced the statistical power of the intervention comparisons. Although our recruitment met the predefined sample size target based on an a priori calculation for detecting moderate effects, the effects observed in the study were smaller than anticipated, suggesting that the study may have been underpowered to detect them. While our study provides valuable preliminary insights, future research should aim to scale up the intervention to a large sample with longer follow-up.

### Recommendations

Our key findings indicate that both interventions in this study had the potential to modify the 24-hour composition of physical behaviours. Specifically, the Reduce sitting at work group experienced behaviour changes due to modifications in the physical environment through the introduction of sit-stand tables equipped with a reminder system. Similarly, the 24-hour group underwent behaviour changes, but their intervention also included an individualized program that targeted behaviours beyond the workplace. However, given that no significant differences were observed between the intervention groups in terms of sitting, standing, and active behaviours, it remains unclear whether the additional behavioural components contributed to greater changes beyond those achieved through workplace environmental modifications alone. While both interventions resulted in some degree of behavioural change, it is important to consider how workplace interventions can better support sustained changes across the entire day [27]. This aligns with recommendations from movement guidelines [30, 64–66], which emphasize the importance of balancing physical behaviours across the entire day, i.e., both during work and leisure hours.

Given that the effect of the intervention may diminish after the initial novelty period wears off—a trend observed in this and other studies [16, 18, 19, 27]—future research should consider incorporating additional strategies to enhance and sustain the intervention’s effectiveness. Such strategies could include using consumer-grade devices to monitor behaviours throughout the day, home-based prompts, home-environmental modifications, or community support groups to encourage activity outside of work hours.

This study provides valuable insights for designing future interventions targeting 24-hour physical behaviour compositions in high-risk populations, such as overweight and obese office workers. Additionally, it offers practical considerations for public health policymakers in low- and middle-income countries, where resources for modifying the physical environment (e.g., providing sit-stand tables) may be limited. In such contexts, alternative approaches that integrate individual- and organizational-level strategies—even if their effects are modest [25, 27, 67]—could still provide meaningful benefits. However, further research is needed to determine the most cost-effective and scalable approaches for sustaining behaviour change across the entire 24-hour period.

### Strengths and limitations

A key strength of the present study is its implementation in a real-life setting, achieving excellent participant retention (0% drop-out rate) and complete data collection at the 3- and 6-month follow-ups. The use of accelerometer-based measurements to monitor time spent in different behaviours is another notable strength, as this type of measurement provides more detailed and accurate data than compared to self-reports [68]. Although participants were blinded to the accelerometer data at the baseline (all groups) and at the 3- and 6-month follow-ups (for the Reduce sitting at work and control groups), some reactivity may have occurred since participants were aware of the purpose of the measurement. Another strength is the use of *Acti4* program to process the accelerometer data and identify physical behaviours with confirmed validity [47, 48]. Additionally, applying a CoDA approach to process the physical behaviour data effectively addresses the inherent co-dependency of physical behaviours that share time within a finite 24-hour window [53, 54].

Some limitations should be recognized when interpreting these results. Data collection was conducted on a convenience sample rather than a random one, which may limit the generalizability of our findings. Additionally, recruitment was restricted to a single city in southeastern Brazil, further constraining the applicability of results to other regions. The participants were employed within a public university and a public university hospital, so our results may not extend to other employment sectors, such as the private and industrial sectors. Because data collection took place during COVID-19 pandemic and due to the nature of our intervention, we included only office workers who were working exclusively at the office, which may limit the relevance of our results for workers in a hybrid model—a common work arrangement in post-pandemic times. While we lacked information on the extent to which participants adhered to public health recommendations during the intervention period, it is worth noting that Brazil primarily relied on recommendations, individual responsibility, and voluntary compliance during this period. Fear of contracting COVID-19 likely discouraged individuals from leaving their homes, which could have influenced behaviours outside the workplace. Finally, we did not collect feasibility or acceptability data (e.g., participant perceptions, adherence, or engagement), which could have provided valuable insights for refining and optimizing the “24-hour time-use approach” in future trials.

## Conclusions

Among Brazilian overweight and obese office workers, the “24-hour time-use approach” may not lead to better improvements in the overall 24-hour behaviours compared to the traditional “reduce sitting at work approach”. Similar results were observed for the domain-specific (i.e., work and leisure) composition of physical behaviours. Additionally, both intervention groups showed changes in their overall 24-hour behaviours compared to the control group, primarily by decreasing sitting and increasing standing, with most changes occurring during work hours.

Changes in behaviours during leisure hours were limited in response to the “24-hour time-use approach”, suggesting that workplace-delivered interventions continue to face challenges in promoting consistent behavioural improvements throughout the entire day. Future interventions may benefit from additional components—such as consumer-grade devices, home-based prompts, and home-environment modifications—to help extend behavioural changes to domains outside the primary workplace setting (e.g., leisure). Given the non-randomised controlled design, small sample size, and short follow-up period, larger, more rigorous studies are needed to confirm these findings and assess the long-term impact of the “24-hour time-use approach”. Finally, we recommend investigating the “24-hour time-use approach” among office workers in hybrid work arrangements, which have become the most common work arrangement since the pandemic.

## Electronic supplementary material

Below is the link to the electronic supplementary material.

Supplementary Material 1

Supplementary Material 2

Supplementary Material 3

## Data Availability

The datasets generated and/or analysed during the current study are not publicly available due to confidentiality of some data sources, but processed data are available from the corresponding author on reasonable request.
