# Supplementary material for: Does the intervention approach matter for improving 24-hour physical behaviours among overweight and obese Brazilian office workers?
Source: BMC Public Health. 2025 Aug 7;25:2699. doi: 10.1186/s12889-025-23957-w (PMC12329870; doi:10.1186/s12889-025-23957-w)
Supplement: Supplementary file 1 — Supplementary Material 1 [file 12889_2025_23957_MOESM1_ESM.docx]

**Additional file**

**Additional file 1.** Set of isometric log-ratio (ilr) coordinates used to address the second aim.

To examine domain-specific effects, the 24-hour behaviour compositions at each measurement point (i.e., baseline, 3- and 6-month follow-ups) were transformed into sets of six orthogonal ilr-coordinates, as exemplified below for work time sitting:

$\mathrm{ilr}_{1}=\sqrt{\frac{6}{7}}\ln\left( \frac{Work sit}{\sqrt[6]{Work stand * Work active * Leisure sit * Leisure stand * Leisure active * Time-in-bed}} \right)$

$\mathrm{ilr}_{2}=\sqrt{\frac{5}{6}}\ln\left( \frac{Work stand}{\sqrt[5]{Work active * Leisure sit * Leisure stand * Leisure active * Time-in-bed}} \right)$

$\mathrm{ilr}_{3}=\sqrt{\frac{4}{5}}\ln\left( \frac{Work active}{\sqrt[4]{Leisure sit * Leisure stand * Leisure active * Time-in-bed}} \right)$

$\mathrm{ilr}_{4}=\sqrt{\frac{3}{4}}\ln\left( \frac{Leisure sit}{\sqrt[3]{Leisure stand * Leisure active * Time-in-bed}} \right)$

$\mathrm{ilr}_{5}=\sqrt{\frac{2}{3}}\ln\left( \frac{Leisure stand}{\sqrt[2]{Leisure active * Time-in-bed}} \right)$

$\mathrm{ilr}_{6}=\sqrt{\frac{1}{2}}\ln\left( \frac{Leisure active}{Time-in-bed} \right)$

In this case, ilr_1_ expresses the ratio of time spent sitting during work to time spent in all other behaviours. For each of the seven behaviours, a separate set of six ilr coordinates was created by rotating the position of behaviours in the equation. This approach allowed each of the seven behaviours to be expressed in relation to the remaining behaviours [1].

**Reference**

1. Dumuid D, Stanford TE, Martin-Fernández JA, Pedišić Ž, Maher CA, Lewis LK, et al. Compositional data analysis for physical activity, sedentary time and sleep research. Stat Methods Med Res. 2018;27:3726–38.
