# Supplementary material for: Does the intervention approach matter for improving 24-hour physical behaviours among overweight and obese Brazilian office workers?
Source: BMC Public Health. 2025 Aug 7;25:2699. doi: 10.1186/s12889-025-23957-w (PMC12329870; doi:10.1186/s12889-025-23957-w)
Supplement: Supplementary file 2 — Supplementary Material 2 [file 12889_2025_23957_MOESM2_ESM.docx]

**Additional file**

**Additional file 2.** Information from valid accelerometry data.

| **Table S1.** Descriptive data on valid accelerometry days for Reduce sitting at work group (intervention with physical environment component), 24-hour group (intervention with physical environment and individual component), and control group. | | | | | |
| --- | --- | --- | --- | --- | --- |
| Group | Evaluation | Total hours | Total days | Mean (SD) hours | Mean (SD; min-max) days |
| Reduce sitting at work | baseline | 1080 | 45 | 72.0 (15.7) | 3.0 (0.7; range 2-5) |
|  | 3-month | 1008 | 42 | 67.2 (13.5) | 2.8 (0.6; range 2-4) |
|  | 6-month | 1032 | 43 | 68.8 (8.4) | 2.9 (0.4; range 2-3) |
| 24-hour | baseline | 1104 | 46 | 73.6 (6.2) | 3.1 (0.3; range 3-4) |
|  | 3-month | 1152 | 48 | 76.8 (13.5) | 3.2 (0.6; range 2-4) |
|  | 6-month | 1224 | 51 | 81.6 (12.2) | 3.4 (0.5; range 3-4) |
| Control | baseline | 1104 | 46 | 73.6 (14.2) | 3.1 (0.6; range 2-4) |
|  | 3-month | 1200 | 50 | 80.0 (14.8) | 3.3 (0.6; range 2-4) |
|  | 6-month | 1200 | 50 | 80.0 (14.8) | 3.3 (0.6; range 2-4) |
| Abbreviations: SD, standard deviation; min, minimum number of valid days measured; max, maximum number of valid days measured. | | | | | |
