# Supplementary material for: Does the intervention approach matter for improving 24-hour physical behaviours among overweight and obese Brazilian office workers?
Source: BMC Public Health. 2025 Aug 7;25:2699. doi: 10.1186/s12889-025-23957-w (PMC12329870; doi:10.1186/s12889-025-23957-w)
Supplement: Supplementary file 3 — Supplementary Material 3 [file 12889_2025_23957_MOESM3_ESM.docx]

**Additional file**

**Additional file 3.** Pair-wise log-ratio variation matrix.

| **Table S1.** Compositional variation matrix for Reduce sitting at work group (intervention with physical environment component), 24-hour group (intervention with physical environment and individual component), and control group. | | | | | |
| --- | --- | --- | --- | --- | --- |
| **Reduce sitting at work group** | | | | | |
| *Baseline* | | | | | |
|  | Sitting | Standing | Active | Time-in-bed | Var-clr (%) |
| Sitting | 0.00 |  |  |  | 0.05 (25%) |
| Standing | 0.14 | 0.00 |  |  | 0.04 (23%) |
| Active | 0.17 | 0.07 | 0.00 |  | 0.05 (28%) |
| Time-in-bed | 0.06 | 0.15 | 0.15 | 0.00 | 0.04 (24%) |
| Total variance |  |  |  |  | 0.18 (100%) |
| *3-month* | | | | | |
|  | Sitting | Standing | Active | Time-in-bed | Var-clr (%) |
| Sitting | 0.00 |  |  | 0.04 | 0.04 (22%) |
| Standing | 0.17 | 0.00 |  | 0.15 | 0.06 (30%) |
| Active | 0.16 | 0.11 | 0.00 | 0.15 | 0.06 (30%) |
| Time-in-bed | 0.04 | 0.15 | 0.15 | 0.00 | 0.04 (18%) |
| Total variance |  |  |  |  | 0.20 (100%) |
| *6-month* | | | | | |
|  | Sitting | Standing | Active | Time-in-bed | Var-clr (%) |
| Sitting | 0.00 |  | 0.10 |  | 0.05 (27%) |
| Standing | 0.18 | 0.00 | 0.09 |  | 0.06 (32%) |
| Active | 0.10 | 0.09 | 0.00 |  | 0.03 (20%) |
| Time-in-bed | 0.07 | 0.12 | 0.12 | 0.00 | 0.04 (21%) |
| Total variance |  |  |  |  | 0.17 (100%) |
| **24-hour group** | | | | | |
| *Baseline* | | | | | |
|  | Sitting | Standing | Active | Time-in-bed | Var-clr (%) |
| Sitting | 0.00 |  |  |  | 0.04 (30%) |
| Standing | 0.14 | 0.00 |  |  | 0.04 (34%) |
| Active | 0.10 | 0.05 | 0.00 |  | 0.02 (18%) |
| Time-in-bed | 0.04 | 0.11 | 0.06 | 0.00 | 0.02 (18%) |
| Total variance |  |  |  |  | 0.13 (100%) |
| *3-month* | | | | | |
|  | Sitting | Standing | Active | Time-in-bed | Var-clr (%) |
| Sitting | 0.00 |  |  |  | 0.04 (30%) |
| Standing | 0.17 | 0.00 |  |  | 0.05 (41%) |
| Active | 0.08 | 0.06 | 0.00 |  | 0.02 (14%) |
| Time-in-bed | 0.03 | 0.11 | 0.06 | 0.00 | 0.02 (15%) |
| Total variance |  |  |  |  | 0.13 (100%) |
| *6-month* | | | | | |
|  | Sitting | Standing | Active | Time-in-bed | Var-clr (%) |
| Sitting | 0.00 |  |  |  | 0.12 (31%) |
| Standing | 0.52 | 0.00 |  |  | 0.16 (43%) |
| Active | 0.26 | 0.15 | 0.00 |  | 0.05 (13%) |
| Time-in-bed | 0.06 | 0.34 | 0.16 | 0.00 | 0.05 (13%) |
| Total variance |  |  |  |  | 0.37 (100%) |
| **Control group** | | | | | |
| *Baseline* | | | | | |
|  | Sitting | Standing | Active | Time-in-bed | Var-clr (%) |
| Sitting | 0.00 |  |  |  | 0.02 (18%) |
| Standing | 0.07 | 0.00 |  |  | 0.02 (23%) |
| Active | 0.10 | 0.06 | 0.00 |  | 0.04 (35%) |
| Time-in-bed | 0.02 | 0.08 | 0.11 | 0.00 | 0.03 (24%) |
| Total variance |  |  |  |  | 0.11 (100%) |
| *3-month* | | | | | |
|  | Sitting | Standing | Active | Time-in-bed | Var-clr (%) |
| Sitting | 0.00 |  |  |  | 0.07 (30%) |
| Standing | 0.25 | 0.00 |  |  | 0.07 (30%) |
| Active | 0.22 | 0.05 | 0.00 |  | 0.05 (23%) |
| Time-in-bed | 0.02 | 0.18 | 0.16 | 0.00 | 0.04 (17%) |
| Total variance |  |  |  |  | 0.22 (100%) |
| *6-month* | | | | | |
|  | Sitting | Standing | Active | Time-in-bed | Var-clr (%) |
| Sitting | 0.00 |  |  |  | 0.03 (24%) |
| Standing | 0.12 | 0.00 |  |  | 0.04 (34%) |
| Active | 0.09 | 0.05 | 0.00 |  | 0.03 (22%) |
| Time-in-bed | 0.03 | 0.11 | 0.08 | 0.00 | 0.02 (21%) |
| Total variance |  |  |  |  | 0.12 (100%) |
| Abbreviations: Total variance = total variance of the composition; Var-clr (%) = absolute and percentage (%) contribution of each behaviour to the total variance.  Values are log-ratios of pair-wise variation. A value close to zero implies that time spent in the corresponding behaviours is nearly proportional; hence there is a high relationship/co-dependence (in proportionality terms) between them. For example, the co-dependence (proportionality) of one behaviour with itself is perfect and the corresponding log-ratio variance (on the diagonal of the variation matrix) is zero. | | | | | |

| **Table S2.** Compositional variation matrix of work (W) and leisure (L) hours for Reduce sitting at work group (intervention with physical environment component), 24-hour group (intervention with physical environment and individual component), and control group. | | | | | | | | |
| --- | --- | --- | --- | --- | --- | --- | --- | --- |
| **Reduce sitting at work group** | | | | | | | | |
| *Baseline* | | | | | | | | |
|  | W-Sitting | W-Standing | W-Active | L-Sitting | L-Standing | L-Active | Time-in-bed | Var-clr (%) |
| W-Sitting | 0.00 |  |  |  |  |  |  | 0.07 (14%) |
| W-Standing | 0.27 | 0.00 |  |  |  |  |  | 0.10 (21%) |
| W-Active | 0.20 | 0.08 | 0.00 |  |  |  |  | 0.05 (12%) |
| L-Sitting | 0.03 | 0.24 | 0.20 | 0.00 |  |  |  | 0.05 (12%) |
| L-Standing | 0.17 | 0.19 | 0.17 | 0.13 | 0.00 |  |  | 0.07 (15%) |
| L-Active | 0.20 | 0.20 | 0.08 | 0.18 | 0.10 | 0.00 |  | 0.07 (15%) |
| Time-in-bed | 0.06 | 0.18 | 0.13 | 0.07 | 0.19 | 0.20 | 0.00 | 0.05 (11%) |
| Total variance | |  |  |  |  |  |  | 0.47 (100%) |
| *3-month* | | | | | | | | |
|  | W-Sitting | W-Standing | W-Active | L-Sitting | L-Standing | L-Active | Time-in-bed | Var-clr (%) |
| W-Sitting | 0.00 |  |  |  |  |  |  | 0.07 (11%) |
| W-Standing | 0.34 | 0.00 |  |  |  |  |  | 0.16 (24%) |
| W-Active | 0.21 | 0.23 | 0.00 |  |  |  |  | 0.11 (17%) |
| L-Sitting | 0.05 | 0.29 | 0.23 | 0.00 |  |  |  | 0.06 (9%) |
| L-Standing | 0.22 | 0.31 | 0.35 | 0.21 | 0.00 |  |  | 0.11 (17%) |
| L-Active | 0.24 | 0.31 | 0.20 | 0.21 | 0.12 | 0.00 |  | 0.09 (14%) |
| Time-in-bed | 0.06 | 0.25 | 0.20 | 0.04 | 0.21 | 0.21 | 0.00 | 0.05 (7%) |
| Total variance | |  |  |  |  |  |  | 0.64 (100%) |
| *6-month* | | | | | | | | |
|  | W-Sitting | W-Standing | W-Active | L-Sitting | L-Standing | L-Active | Time-in-bed | Var-clr (%) |
| W-Sitting | 0.00 |  |  |  |  |  |  | 0.08 (13%) |
| W-Standing | 0.40 | 0.00 |  |  |  |  |  | 0.17 (24%) |
| W-Active | 0.15 | 0.15 | 0.00 |  |  |  |  | 0.07 (11%) |
| L-Sitting | 0.10 | 0.32 | 0.20 | 0.00 |  |  |  | 0.07 (10%) |
| L-Standing | 0.29 | 0.37 | 0.34 | 0.24 | 0.00 |  |  | 0.13 (19%) |
| L-Active | 0.23 | 0.39 | 0.25 | 0.20 | 0.12 | 0.00 |  | 0.11 (16%) |
| Time-in-bed | 0.10 | 0.20 | 0.10 | 0.10 | 0.23 | 0.24 | 0.00 | 0.04 (6%) |
| Total variance | |  |  |  |  |  |  | 0.68 (100%) |
| **24-hour group** | | | | | | | | |
| *Baseline* | | | | | | | | |
|  | W-Sitting | W-Standing | W-Active | L-Sitting | L-Standing | L-Active | Time-in-bed | Var-clr (%) |
| W-Sitting | 0.00 |  |  |  |  |  |  | 0.07 (9%) |
| W-Standing | 0.51 | 0.00 |  |  |  |  |  | 0.27 (33%) |
| W-Moving | 0.34 | 0.16 | 0.00 |  |  |  |  | 0.15 (19%) |
| L-Sitting | 0.06 | 0.42 | 0.28 | 0.00 |  |  |  | 0.06 (7%) |
| L-Standing | 0.21 | 0.62 | 0.47 | 0.26 | 0.00 |  |  | 0.15 (18%) |
| L-Moving | 0.12 | 0.58 | 0.38 | 0.13 | 0.07 | 0.00 |  | 0.08 (10%) |
| Time-in-bed | 0.06 | 0.42 | 0.23 | 0.05 | 0.20 | 0.11 | 0.00 | 0.04 (4%) |
| Total variance | |  |  |  |  |  |  | 0.81 (100%) |
| *3-month* | | | | | | | | |
|  | W-Sitting | W-Standing | W-Active | L-Sitting | L-Standing | L-Active | Time-in-bed | Var-clr (%) |
| W-Sitting | 0.00 |  |  |  |  |  |  | 0.05 (10%) |
| W-Standing | 0.32 | 0.00 |  |  |  |  |  | 0.13 (27%) |
| W-Moving | 0.20 | 0.13 | 0.00 |  |  |  |  | 0.11 (22%) |
| L-Sitting | 0.05 | 0.29 | 0.23 | 0.00 |  |  |  | 0.05 (11%) |
| L-Standing | 0.12 | 0.22 | 0.28 | 0.12 | 0.00 |  |  | 0.07 (14%) |
| L-Moving | 0.10 | 0.22 | 0.25 | 0.11 | 0.07 | 0.00 |  | 0.05 (11%) |
| Time-in-bed | 0.03 | 0.20 | 0.15 | 0.07 | 0.12 | 0.10 | 0.00 | 0.03 (6%) |
| Total variance | |  |  |  |  |  |  | 0.48 (100%) |
| *6-month* | | | | | | | | |
|  | W-Sitting | W-Standing | W-Active | L-Sitting | L-Standing | L-Active | Time-in-bed | Var-clr (%) |
| W-Sitting | 0.00 |  |  |  |  |  |  | 0.13 (14%) |
| W-Standing | 0.70 | 0.00 |  |  |  |  |  | 0.27 (29%) |
| W-Moving | 0.26 | 0.38 | 0.00 |  |  |  |  | 0.11 (11%) |
| L-Sitting | 0.05 | 0.75 | 0.29 | 0.00 |  |  |  | 0.15 (16%) |
| L-Standing | 0.42 | 0.24 | 0.36 | 0.46 | 0.00 |  |  | 0.13 (14%) |
| L-Moving | 0.38 | 0.31 | 0.26 | 0.34 | 0.09 | 0.00 |  | 0.10 (11%) |
| Time-in-bed | 0.05 | 0.48 | 0.16 | 0.11 | 0.30 | 0.26 | 0.00 | 0.06 (6%) |
| Total variance | |  |  |  |  |  |  | 0.95 (100%) |
| **Control group** | | | | | | | | |
| *Baseline* | | | | | | | | |
|  | W-Sitting | W-Standing | W-Active | L-Sitting | L-Standing | L-Active | Time-in-bed | Var-clr (%) |
| W-Sitting | 0.00 |  |  |  |  |  |  | 0.05 (12%) |
| W-Standing | 0.25 | 0.00 |  |  |  |  |  | 0.11 (28%) |
| W-Active | 0.18 | 0.16 | 0.00 |  |  |  |  | 0.07 (17%) |
| L-Sitting | 0.11 | 0.15 | 0.18 | 0.00 |  |  |  | 0.05 (14%) |
| L-Standing | 0.08 | 0.19 | 0.13 | 0.10 | 0.00 |  |  | 0.03 (9%) |
| L-Active | 0.09 | 0.21 | 0.10 | 0.17 | 0.06 | 0.00 |  | 0.05 (12%) |
| Time-in-bed | 0.04 | 0.21 | 0.13 | 0.07 | 0.08 | 0.11 | 0.00 | 0.03 (8%) |
| Total variance | |  |  |  |  |  |  | 0.40 (100%) |
| *3-month* | | | | | | | | |
|  | W-Sitting | W-Standing | W-Active | L-Sitting | L-Standing | L-Active | Time-in-bed | Var-clr (%) |
| W-Sitting | 0.00 |  |  |  |  |  |  | 0.08 (12%) |
| W-Standing | 0.41 | 0.00 |  |  |  |  |  | 0.17 (26%) |
| W-Active | 0.23 | 0.13 | 0.00 |  |  |  |  | 0.08 (12%) |
| L-Sitting | 0.12 | 0.45 | 0.37 | 0.00 |  |  |  | 0.14 (21%) |
| L-Standing | 0.20 | 0.22 | 0.13 | 0.29 | 0.00 |  |  | 0.06 (9%) |
| L-Active | 0.23 | 0.33 | 0.18 | 0.31 | 0.07 | 0.00 |  | 0.09 (14%) |
| Time-in-bed | 0.02 | 0.32 | 0.19 | 0.10 | 0.16 | 0.18 | 0.00 | 0.04 (7%) |
| Total variance | |  |  |  |  |  |  | 0.66 (100%) |
| *6-month* | | | | | | | | |
|  | W-Sitting | W-Standing | W-Active | L-Sitting | L-Standing | L-Active | Time-in-bed | Var-clr (%) |
| W-Sitting | 0.00 |  |  |  |  |  |  | 0.05 (12%) |
| W-Standing | 0.23 | 0.00 |  |  |  |  |  | 0.11 (25%) |
| W-Active | 0.05 | 0.22 | 0.00 |  |  |  |  | 0.04 (9%) |
| L-Sitting | 0.09 | 0.15 | 0.11 | 0.00 |  |  |  | 0.05 (12%) |
| L-Standing | 0.19 | 0.20 | 0.13 | 0.19 | 0.00 |  |  | 0.07 (17%) |
| L-Active | 0.21 | 0.18 | 0.14 | 0.14 | 0.09 | 0.00 |  | 0.07 (17%) |
| Time-in-bed | 0.02 | 0.19 | 0.04 | 0.10 | 0.14 | 0.17 | 0.00 | 0.03 (8%) |
| Total variance | |  |  |  |  |  |  | 0.43 (100%) |
| Total variance = total variance of the composition; Var-clr (%) = absolute and percentage (%) contribution of each behaviour to the total variance.  Values are log-ratios of pair-wise variation. A value close to zero implies that time spent in the corresponding behaviours is nearly proportional; hence there is a high relationship/co-dependence (in proportionality terms) between them. For example, the co-dependence (proportionality) of one behaviour with itself is perfect and the corresponding log-ratio variance (on the diagonal of the variation matrix) is zero. | | | | | | | | |
